# Benzoylphenyl thiocyanates are new, effective inhibitors of the mycobacterial resuscitation promoting factor B protein

**DOI:** 10.1186/s12941-017-0244-7

**Published:** 2017-11-02

**Authors:** Galina R. Demina, Vadim D. Nikitushkin, Margarita O. Shleeva, Olga B. Riabova, Alexander Yu. Lepioshkin, Vadim A. Makarov, Arseny S. Kaprelyants

**Affiliations:** 1 Laboratory of Biochemistry of Stress in Microorganisms, A.N. Bach Institute of Biochemistry, Federal Research Centre “Fundamentals of Biotechnology” of the Russian Academy of Sciences, Leninsky prospect, 33 (2), Moscow, 119071 Russia; 2 Laboratory of Biomedicinal Chemistry, A.N. Bach Institute of Biochemistry, Federal Research Centre “Fundamentals of Biotechnology” of the Russian Academy of Sciences, Leninsky prospect, 33 (2), Moscow, 119071 Russia

**Keywords:** Benzoylphenyl thiocyanates, Resuscitation promoting factor (Rpf), Dormant mycobacteria, *Mycobacterium tuberculosis*, Drug design

## Abstract

**Background:**

Resuscitation promoting factors (Rpfs) are the proteins involved in the process of reactivation of the dormant cells of mycobacteria. Recently a new class of nitrophenylthiocyanates (NPTs), capable of inhibiting the biological and enzymatic activities of Rpfs has been discovered. In the current study the inhibitory properties of the compounds containing both nitro and thiocyanate groups alongside with the compounds with the modified number and different spatial location of the substituents are compared.

**Methods:**

New benzoylphenyl thiocyanates alongside with nitrophenylthiocyanates were tested in the enzymatic assay of bacterial peptidoglycan hydrolysis as well as against strains of several actinobacteria (*Mycobacterium smegmatis, Mycobacterium tuberculosis*) on in-lab developed models of resuscitation of the dormant forms.

**Results:**

Introduction of the additional nitro and thiocyanate groups to the benzophenone scaffold did not influence the inhibitory activity of the compounds. Removal of the nitro groups analogously did not impair the functional properties of the molecules. Among the tested compounds two molecules without nitro group: 3-benzoylphenyl thiocyanate and 4-benzoylphenyl thiocyanate demonstrated the maximum activity in both enzymatic assay (inhibition of the Rpf-mediated peptidoglycan hydrolysis) and in the resuscitation assay of the dormant *M. tuberculosis* cells.

**Conclusions:**

The current study demonstrates dispensability of the nitro group in the NPT’s structure for inhibition of the enzymatic and biological activities of the Rpf protein molecules. These findings provide new prospects in anti-TB drug discovery especially in finding of molecular scaffolds effective for the latent infection treatment.

**Electronic supplementary material:**

The online version of this article (10.1186/s12941-017-0244-7) contains supplementary material, which is available to authorized users.

## Background

Tuberculosis is one of the most widespread and dangerous disease, whose causative agent *Mycobacterium tuberculosis* (MTB) is able to pass into a specific state of dormancy under stress conditions [1]. One of the main features of the dormancy, in addition to the lowered metabolic activity, is the decrease in sensitivity to the commonly used antibiotics effective against the active form of MTB [2]. The pathogen can survive in a host in a latent, quiescent, asymptomatic state, maintaining the possibility of transition into the metabolically active state, provoking the acute disease development. Resuscitation promoting factors (Rpfs) belong to the protein family encoded in a number of actinobacteria (including MTB), are known to be involved in the process of reactivation of the dormant mycobacterial cells [3]. Elucidation of the structural organization of the Rpf proteins [4–7], as well as the discovery of their enzymatic activity [8, 9] allows considering this protein as a promising molecular target for screening and development of scaffolds, capable of suppressing the reactivation process of the dormant mycobacterial forms (among them MTB), what would potentially pave a novel way toward creation of new promising drugs with anti-TB activity, including ones with the activity against latent TB.

Recently a screening aimed at identifying of some compounds, that would be potentially able to inhibit enzymatic and biological activities of Rpfs, has been carried out, and a new class of nitrophenylthiocyanates (NPT) was found [10]. NPTs is a diverse panel of aromatic compounds, containing a combination of nitro and thiocyanate groups, among them one inhibitor: 4-benzoyl-2-nitrophenyl thiocyanate (I) was the most effective [10]. In the previous work of Ruggiero with co-authors [11], a combination of X-ray crystallography and a computational approach (REMD with the calculation of free energy of binding) allowed to investigate the mechanism of protein-ligand (I) interaction, having suggested the importance of the thiocyanate group for inhibitory properties of the 4-benzoyl-2-nitrophenyl thiocyanate. These findings also suggested that the nitro group in the NPT’s structure was presumable dispensable for their activity [11].

The purpose of the current study is to verify the latter assumption experimentally by synthesizing of a new panel of NPT derivatives—benzoylphenyl thiocyanates (BPTs), with the subsequent comparison of the inhibitory properties of these compounds with their progenitors in the in-lab developed enzymatic and biological assays.

## Methods

### Chemistry

All reagents and solvents were purchased from commercial suppliers and used without further purification. Melting points were determined using Electrothermal 9001 analyzer and are uncorrected. ^1^H NMR spectra were recorded at 400 MHz on Varian Unity + 400. Shifts for NMR are reported in ppm downfield from TMS (s). A Waters Micromass ZQ detector was used in ESI MS for identification of various products. Elemental analyses were carried out using a C,H,F,N elemental analyzer, model Carlo-Erba 5500 instrument. The results are within ± 0.3% of the theoretical values. Merck silica gel 60 F254 plates were used for analytical TLC; column chromatography was performed on Merck silica gel 60 (70–230 mesh).

#### 4-Benzoyl-2-nitrophenyl thiocyanate (I)

Solution of 4 g (15.3 mM) of (4-chloro-3-nitrophenyl)(phenyl)methanone in 15 ml of NMP was treated by 1.63 g (16.8 mM) potassium thiocyanate and the reaction mixture was heated for 7 h at 150 °C. The solution was cooled and diluted by water. Formed oil layer was separated and treated by ethanol. The solid was filtered off and crystallized from EtOH. The yield of (I) was 1.65 g (38%). Mp 132–136 °C. Mass (EI), *m/z* (*I*_*relat*_.(%)): 284.2911 [M]^+^ (62). C_14_H_8_N_2_O_3_S. ^1^H NMR (DMSO-d_6_): 8.41 (d, 1H, *J* = 9.2 Hz, CH), 8.02 (s, 1H, CH), 7.69 (m, 3H, 3CH) and 7.41 (m, 3H, 3CH) ppm.

#### Bis(4-thiocyano-3-nitrophenyl)methanone (IV)

Solution of 2 g (5.86 mM) of bis(4-chloro-3-nitrophenyl)methanone in 25 ml of acetone was treated by 1.2 g (12.0 mM) potassium thiocyanate and the reaction mixture was refluxed for 2 h. Formed suspension was filtered off and filtrate was diluted by water; yellow precipitate was filtered off and crystallized from DMF/EtOH. The yield of (IV) was 1.78 g (78%). Mp 192–195 °C. Mass (EI), *m/z* (*I*_*relat*_.(%)): 386.3641 [M]^+^ (39). C_15_H_6_N_4_O_5_S_2_. ^1^H NMR (DMSO-d_6_): 8.46 (d, 2H, *J* = 9.1 Hz, 2CH), 7.97 (s, 2H, 2CH) and 7.70 (d, 2H, *J* = 9.1 Hz, 2CH) ppm.

### General procedure of benzoylphenyl thiocyanates (BPT) synthesis

Solution of 1 mM of sodium nitrite in 2 ml of water was slowly added at 0 °C to the suspension of corresponding *R*-aminophenyl(phenyl)methanone (1 mM) in 15 ml of 10% hydrochloric acid. The reaction mixture was held at 0 °C for 15 min and was slowly added to the in advance prepared suspension of 3 mM of potassium thiocyanate and 2 mM of copper (I) thiocyatate in 5 ml of water. The suspension was left at room temperature for 3 h and diluted by water. The precipitate was filtered off and the filtrate was extracted by ethylacetate (3 × 30 ml). Combined extracts were dried by sodium sulfate, filtered off trough silica gel cushion and evaporated. The residue oil layer was purified by column chromatography on silica gel (hexan:acetone 4/1).

#### 3-Benzoylphenyl thiocyanate (II)

The yield 47%. Mp 167–169 °C. Mass (EI), *m/z* (*I*_*relat*_.(%)): 239.2936 [M]^+^ (57). C_14_H_9_NOS. ^1^H NMR (DMSO-d_6_): 7.99 (s, 1H, CH), 7.76–7.36 (m, 8H, 8CH) ppm.

#### 4-Benzoylphenyl thiocyanate (III)

The yield 68%. Mp 143–146 °C. Mass (EI), *m/z* (*I*_*relat*_.(%)): 239.2934 [M]^+^ (33). C_14_H_9_NOS. ^1^H NMR (DMSO-d_6_): 7.79 (br m, 4H, 4CH), 7.55–7.41 (m, 5H, 5CH) ppm.

#### Bis(4-thiocyanophenyl)methanone (V)

The yield 32%. Mp 171–173 °C. Mass (EI), *m/z* (*I*_*relat*_.(%)): 296.3689 [M]^+^ (45). C_15_H_8_N_2_OS_2_. ^1^H NMR (DMSO-d_6_): 7.78 (d, 4H, *J* = 15.8 Hz, 4CH), 7.53 (d, 4H, *J* = 15.8 Hz, 4CH) ppm.

#### Bis(3-thiocyanophenyl)methanone (VI)

The yield 38%. Mp 164–167 °C. Mass (EI), *m/z* (*I*_*relat*_.(%)): 296.3687 [M]^+^ (72). C_15_H_8_N_2_OS_2_. ^1^H NMR (DMSO-d_6_): 7.95 (s, 2H, 2CH), 7.70 (m, 2H, 2CH), 7.63 (m, 2H, 2CH) and 7.51 (m, 2H, 2CH) ppm.

### Bacterial strains and growth conditions

*Micrococcus luteus*, fleming strain 2665, was grown in liquid rich medium (NB, Himedia) on an orbital shaker at 30 °C.

*Mycobacterium smegmatis* mc^2^155 was grown in Sauton’s liquid medium on an orbital shaker at 37 °C.

*Mycobacterium tuberculosis* H37Rv was grown under agitation at 37 °C (200 rpm), in Sauton’s medium (supplemented by ADC with 0.05% w/v Tween 80 as described [12].

### MIC determination

Determination of the MICs was performed according to the in-lab developed assays [10], based on the CLSI protocols [13]. *M. luteus* cells were grown up to the stationary phase in the rich medium (NB, Himedia) for 48 h. The culture was washed with the sterile medium thrice with subsequent dilutions down to a finite cell concentration of 10^7^ cells/ml. The diluted cells were distributed into 96-well plates (250 ml per well) and the test compounds at the concentrations of 2.5–100 µM were added. Appropriate cells’ dilution without the inhibitors added were serving as a control. The kinetics of *M. luteus* growth was recorded in Multiskan Analyzer (Thermo, Finland) (620 nm filter; 30 °C for 24 h with shaking).

The inoculum of *M. smegmatis* was grown in 2× rich medium (NB, Himedia) supplemented with 0.05% Tween-80 for 16 h. 10^7^ cells/ml were dispensed into 96-well plates (300 ml per well) with the addition of 0.1% Tween-80 and tested compounds were transferred to each microplate well at the concentrations ranging from 2.5 to 100 µM.

The kinetics of *M. smegmatis* growth was recorded using a Multiskan Analyzer (Thermo, Finland) (620 nm filter; 37 °C, 28 h with shaking).

*Mycobacterium tuberculosis* was grown for 8 days in Sauton’s medium supplemented with ADC in the presence of 0.05% Tween-80. Cells were inoculated into 10 ml of the fresh medium (to reach the concentration of 10^6^ cells/ml) in 50 ml screw test tubes. Concentrations of inhibitors varied over the range of 25–100 µM. The test tubes were incubated at 37 °C without shaking; OD_600_ was measured periodically (for 12 days) using an Eppendorf BioPhotometer (Germany). All MIC determination test were repeated three folds in separate times.

### Formation of the “nonculturable” (NC), dormant cells of *M. tuberculosis*

NC, dormant forms of MTB were obtained by the method of gradual self-acidification of the culture grown on modified Sauton’s medium up to 3.5 months [14]. These cells were characterized by complete nonculturability with CFU < 10^1^ on solid medium.

### Resuscitation procedure

NC *M. tuberculosis* cells were separated from the spent medium by centrifugation for 20 min, 5000*g* and inoculated into the twice diluted Sauton’s medium containing ADC (1:10). Resuscitation of NC *M. tuberculosis* cells was performed in 15 ml screw test tubes containing serially diluted dormant cells in 2 ml of Sauton’s medium. Each tube was supplemented with ADC (1:10) with or without inhibitors at the concentrations of 25–50 µM. Tubes were incubated for 40–60 days without shaking at 37 °C until the number of the tubes with visible growth was stabilized. The number of resuscitated, viable cells was determined by the “most probable number method” (MPN) [15].

### Viability evaluation by CFU

Bacterial suspensions were serially diluted in fresh Sauton’s medium, three replicates of 100 μl samples from each dilution were spotted on NBE agar. Plates were incubated at 37 °C for 5 days followed by evaluation of the CFU number. The lower limit of detection was 10^1^ CFU/ml.

### Peptidoglycan (PG) preparation, labelling and Rpf’s enzymatic activity estimation

Peptidoglycan of *M. smegmatis* was obtained according to the previously published protocol [16].

To isolate and to purify PG from MTB the procedure was modified as follows:

*M. tuberculosis* H37Rv was grown on Sauton’s medium (pH 7.0) at 37 °C until the cells reached the late logarithmic phase (~ 8 days).

Cells were collected by centrifugation (4000*g*, for 30 min), washed three times with 0.9% NaCl, autoclaved (121 °C, 20 min) and resuspended in the breaking buffer (2% w/v Triton X-100 in PBS (0.1 M KH_2_PO_4_, 0.01 M NaCl, pH 7.4). The bacterial suspension was mechanically disrupted using a BeadBeater (Biospec Products, USA) with zirconium beads for 10 rounds of beating (for 1 min each).

Undestroyed cells and beads were removed by centrifugation at 4000*g* for 10 min. Broken cells were left overnight in the breaking buffer to extract the remaining soluble material. Then cells were carefully washed from the detergent. To remove the associated proteins, the pelleted cell wall material was extracted five times with 2% SDS in PBS at 95 °C for 1 h. The treated pellet was washed with water, 80% acetone in water, pure acetone and then lyophilized to yield the purified cell walls (rich in mycolic acids, arabinogalactan, and PG).

Mycolic acids were removed by saponification—refluxing with 0.5% KOH in methanol/toluene solution (1:1) for 96 h. The insoluble residue was collected by centrifugation (10,000*g* for 5 min). The pellet was washed twice with methanol and twice with diethyl ether before freeze-drying. To remove arabinogalactan from the arabinogalactan—PG complex, the cell wall sample was incubated in the 0.1 M H_2_SO_4_ at 37 °C for 7 days [17]. The obtained PG was washed five times with water and lyophilized. The final PG samples were kept at 4 °C. FITC labelling of PG was performed according [16, 18]. Enzymatic activity of the protein was assessed by the level of the released florescent PG fragments according to the previous paper [16]. The concentration range of the compounds was 0.35–35 µM.

### Isolation and purification of the recombinant RpfB

The catalytic domain of the RpfB protein (residues 280–362) was isolated from the *E. coli* producer strain (kanamycin-resistant), according to the previously published protocol [19] in our modification [16].

## Results

**Fig. 1:**
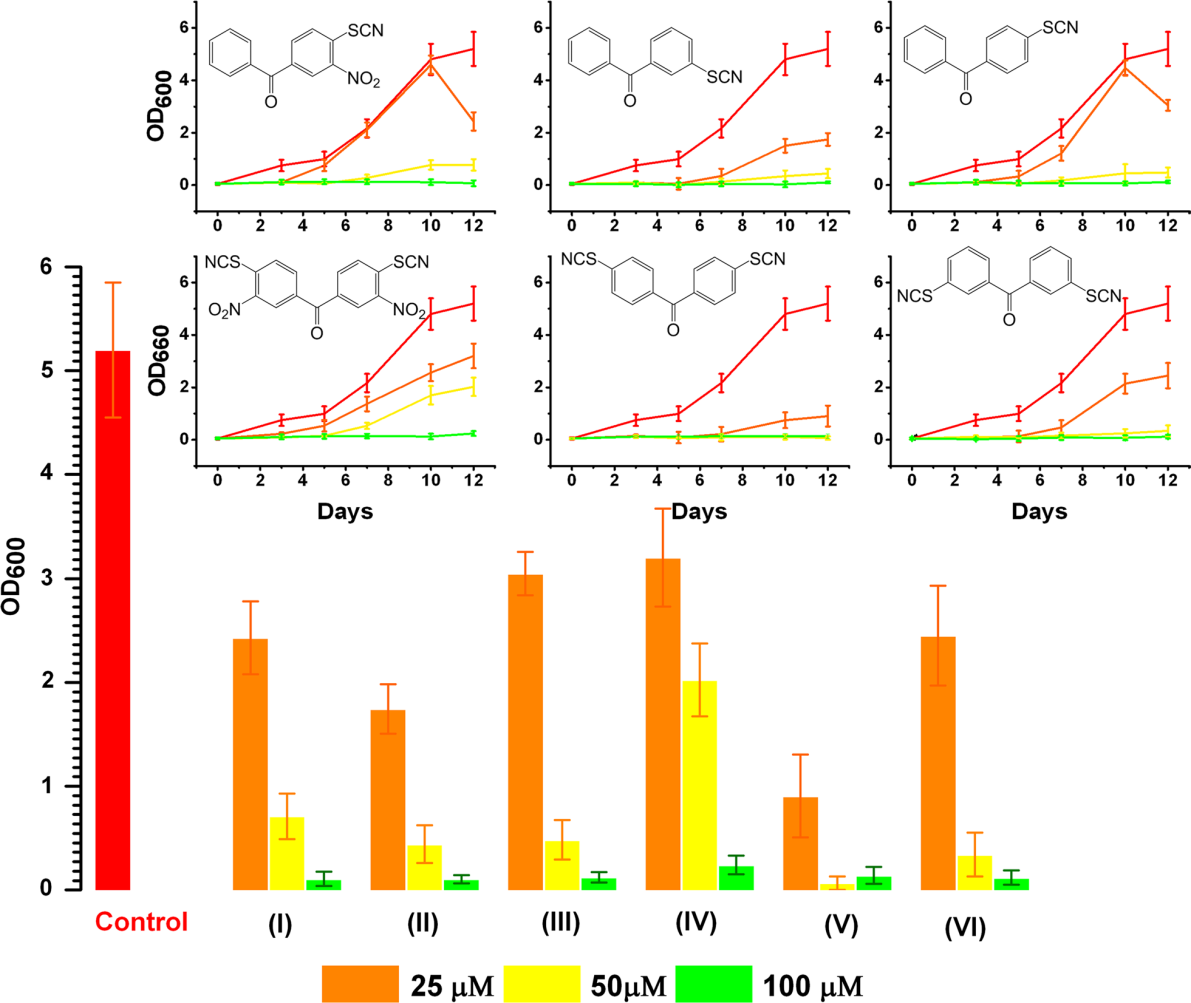
Influence of the NPT and BPT compounds on active growth of *M. tuberculosis.* MTB cells were grown for 12 days as described in the “Methods” section. The histogram shows inhibitory effect of the compounds tested at the 12th day from the beginning of the experiment. In the *Inset,* the growth curves in the presence of the inhibitors and control (without addition of the compounds) are shown. The compounds were added at the concentrations of 25, 50, 100 µM in triplicates (shown in different colors). The error bars represent the standard deviation of the measurements. The Roman numerals in the brackets correspond to the compounds’ numbers listed in the Table 1

For the current study, a panel of 4-benzoyl-2-nitrophenyl thiocyanate derivatives has been designed by the methods of medicinal chemistry and successfully synthesized. The newly created chemicals were varying in a number of thiocyanate and nitro groups, as well as in their mutual spatial position: among them were benzoylphenyl thiocyanates (BPTs): (II)–(III), (V)–(VI) and a nitrophenylthiocyanate (NPT): (IV) (Table 1, Fig. 1). All the compounds were tested for their inhibitory activity on different screening models. The main criterion for the potential efficiency of the selected compounds was chosen their ability to suppress enzymatic activity of the catalytic domain of RpfB.

**Table 1:** Inhibitory effect of the NPT and BPT compounds on the Rpf-mediated hydrolysis of mycobacterial peptidoglycan and on bacterial growth

№	Chemical name	Formula	IC_50_ Rpf-hydrolysis of PG, µM		MIC of the compound, µM	
			*M. smegmatis*	*M. tuberculosis*	*M. luteus*	*M. smegmatis*
**(I)**	4-Benzoyl-2-nitrophenyl thiocyanate		2.4 ± 0.7	4.0 ± 2.1	25–35	18–35
**(II)**	3-Benzoylphenyl thiocyanate		0.4 ± 0.0	1.7 ± 0.4	32–42	32–42
**(III)**	4-Benzoylphenyl thiocyanate		0.9 ± 0.5	1.2 ± 0.5	25–32	21–42
**(IV)**	Bis(4-thiocyano-3-nitrophenyl)methanone		3.7 ± 0.2	5.8 ± 2.0	47–52	26–52
**(V)**	Bis(4-thiocyanophenyl)methanone		2.5 ± 0.6	4.3 ± 2.4	48–51	34
**(VI)**	Bis(3-thiocyanophenyl)methanone		2.0 ± 0.8	3.6 ± 1.2	34–41	34

Enzymatic activity was calculated as a value of the half maximal inhibitory concentration (IC_50_). IC_50_ values were calculated in the enzymatic assay of the Rpf-mediated PG hydrolysis (concentration of RpfB_280–326_ was 10 μg/ml). The experiment was repeated three times with nearly identical results (mean values ± SD are shown)

MIC was evaluated as the minimal inhibitory concentration of the compounds sufficient for suppression of active growth of the bacteria. The experiments were repeated at least three times with nearly identical results (mean values ± SD are shown)

Thus, in the enzymatic assay, the new compounds were tested for their ability to inhibit the process of the Rpf-mediated hydrolysis of the FITC-labeled mycobacterial PG isolated from both bacterial cultures: *M. smegmatis* and *M. tuberculosis* [16, 20]. The level of hydrolysis was estimated by measuring the amount of the FITC-labeled fragments of peptidoglycan released into supernatant [16, 20].

It was found that the inhibitory activity of the compounds lacking the nitro groups was several times higher than the activity of the control (I), given at the same concentration (Table 1).

Followed by the enzymatic assay, all the compounds underwent an examination on their ability to suppress active growth of *M. luteus* (a single Rpf protein in this species is essential for replication [21]) and *M. smegmatis* (a faster growing, non-pathogenic close relative of MTB, containing four *rpf* genes).

The minimum inhibitory concentrations (MICs) of the synthesized compounds required for complete growth suppression of both bacterial cultures were determined on the liquid medium in a concentration range of 1–100 µM.

Admittedly, the tested compounds were, generally, slightly less efficient than NPTs, however, one leading compound (III, BPT) was very close to the (I, NPT) (Table 1). All the tested compounds revealed similar values of the MICs encompassed within the following ranges: 25–52 µM for *M. luteus* and 18–52 µM for *M. smegmatis*.

Compounds at the concentrations of 25, 50 and 100 µM were also tested for their in vitro activity against growing *M. tuberculosis* H37Rv cells.

BPTs lacking the nitro functional groups (II), (V), (VI) suppressed MTB growth at the concentrations close to the MICs found for *M. luteus* and *M. smegmatis*. The suppressory efficacy of these derivatives tested at 50 µM appeared to be even higher than for the compounds, containing the nitro group in their structure (Fig. 1).

A complete growth suppression (98–99%), however, becomes apparent at the concentration of 100 µM.

**Fig. 2:**
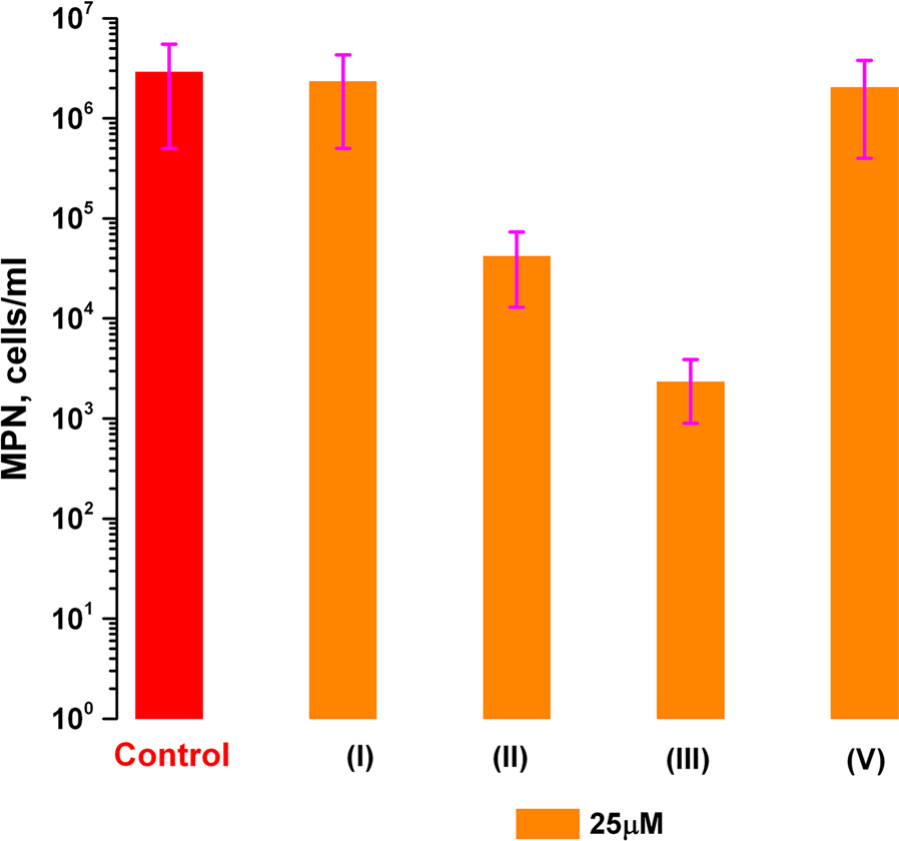
Influence of the NPT and BPT compounds on suppression of resuscitation of the dormant *M. tuberculosis* cells. Resuscitation process was evaluated by standard MPN assay (for details see the “Methods”). The compounds were tested at the concentration of 25 µM in three replicates. Plates were incubated for 40–60 days without shaking at 37 °C. The error bars represent the standard deviation of the measurements. The Roman numerals in the brackets correspond to the compounds’ numbers listed in the Table 1

The selected BPTs were examined for their ability to suppress reactivation of the NC dormant forms of MTB (Fig. 2). Since the synthesized derivatives were shown to suppress growth of the active cells, we used the concentration of 25 µM, what could give a chance to the bacterial culture to develop visible OD (necessary for the MPN evaluation).

The number of the reactivated from dormancy cells was estimated by the MPN assay [10].

From the Fig. 2, the most potent candidates appeared to be the compounds containing a single thiocyanate group, substituted at *meta*- and *para*-position toward the benzophenone core—(II) and (III) respectively. Obviously, the compounds lacking the nitro groups demonstrate noticeable suppressive effect on TB reactivation.

## Discussion

Having adopted a protocol of protein ligand docking based on the Replica-Exchange Molecular Dynamics (REMD) technique, Ruggiero with co-authors have established that the main contribution in the protein-ligand interaction was made by hydrophobic (aromatic) interactions between the phenyl rings of (I) and aromatic amino acids (Tyr305, Trp352, Phe311) building up the catalytic cleft of the enzyme. The observed hydrophobic interactions indicate rather unspecific binding of the NPTs to the RpfB catalytic site [11].

The specific interactions (formation of hydrogen bonds) were mediated primarily by the thiocyanate functional group of NPT, which was found to interact with the amino acid residues of the catalytic cleft (among them Asp312, Glu 292), whereas the nitro group has been found not to contribute to binding at all [11]. These results suggested that the thiocyanate group bound to the phenyl ring (and aryls of the benzophenone by themselves) are the key components for inactivation of the catalytic activity of the RpfB molecule. This assumption was subjected to the experimental check in the current study. Support for this assumption may be found in the analysis of the results obtained in the enzymatic and biological assays. The enzymatic assay revealed potential ability of the tested compounds to inhibit enzymatic activity of the Rpf protein, favoring this hypothesis.

The biological effect of the newly synthesized compounds was tested on the Rpf-producing bacteria: *M. luteus, M. smegmatis,* judging by suppression of active growth in the liquid medium. It was found that BPTs inhibited growth of *M. luteus* similar to NPTs (owing to the essentiality of the *rpf* gene for this bacterium). BPTs and NPTs were also shown to suppress growth of *M. smegmatis* (information on essentiality of the corresponding genes is lacking). Some of the BPTs (II), (V), (VI) revealed moderate inhibitory effect on MTB growth in the liquid medium. This effect is in apparent contradiction to the found dispensability of the RpfA-E proteins for *M. tuberculosis* growth in vitro [22–25]. However, MTB apparently encodes other lytic transglycosylases differing from Rpfs [26–28], that may provide a basis for the observed inhibitory activity.

Comparison of the data on inhibitory activity of the tested compounds (Table 1, Fig. 2) demonstrates strong correlation between the effectiveness of hydrolytic activity inhibition and the process of resuscitation, underlying the significance of Rpfs in resuscitation of the dormant MTB cells. In both experiments, compounds lacking the nitro groups manifested the same level of potency, shown for NPTs, or sometimes even higher (II), (III), justifying the stated hypothesis experimentally.

**Fig. 3:**
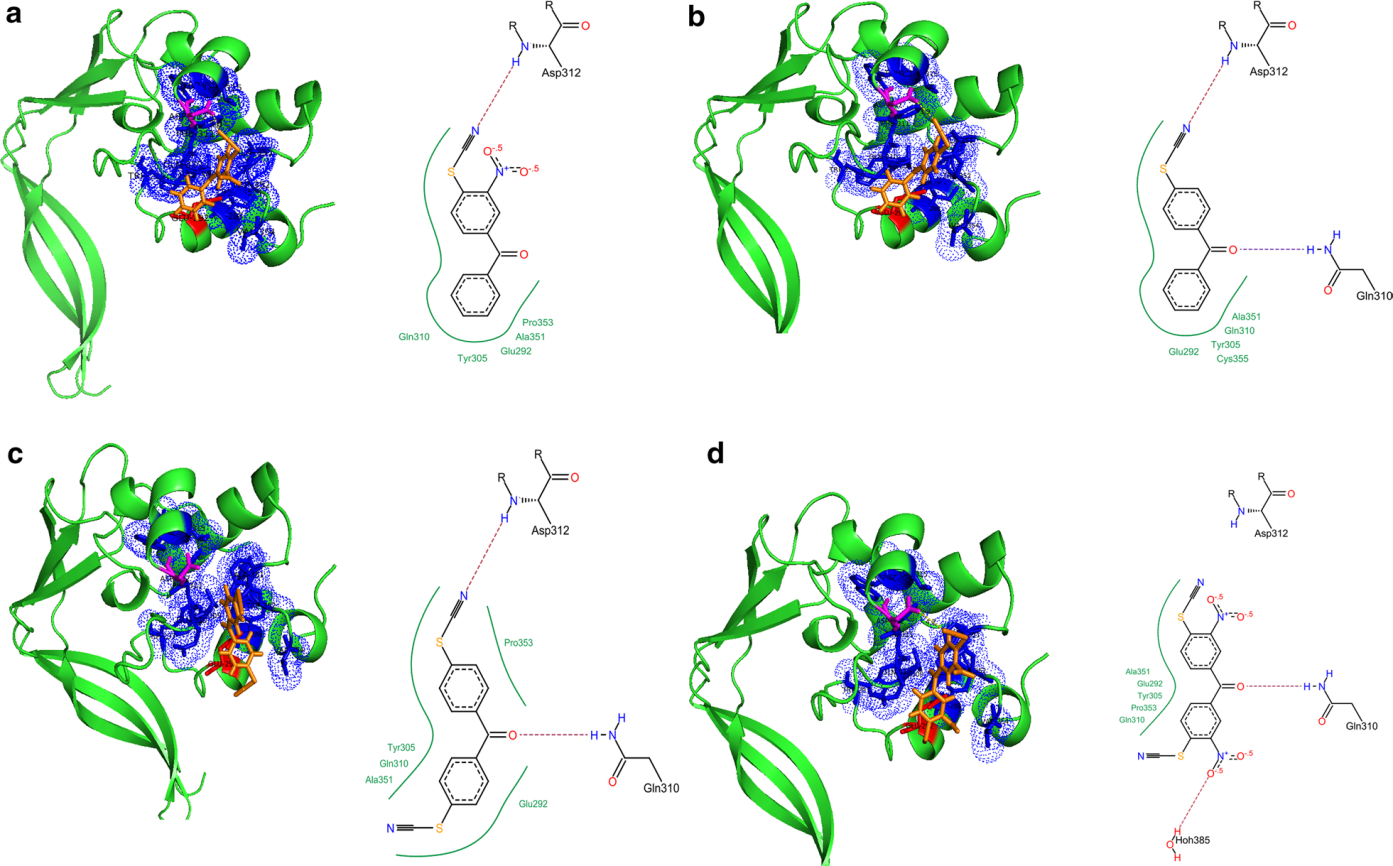
Predictive analysis of the interactions of some NPTs and BPTs with the catalytic domain of RpfB (3EO5) based on the Flexx docking procedure. **a** Compound (I) was predicted to interact with the hydrophobic surrounding of the catalytic cleft of the protein molecules, whereby the thiocyanate group was shown to be able to interact with Asp 312; the nitro group was shown to be located on the surface of the molecule, not contributing to the inhibitory properties of the molecule (∆G ≈ − 7 kJ/mol); **b** compound (III) an analogous compound, completely lacking the nitro groups was shown to interact with the catalytic cleft of the protein similarly to the afore described mechanism for (I) (∆G ≈ − 8 kJ/mol); **c** compound (V) containing the double set of the thiocyanate groups substituted at the para-position toward the benzophenone group was predicted to interact with the catalytic cleft of the RpfB molecule mainly through hydrophobic interactions between the benzophenone rings and hydrophobic surroundings of the catalytic cleft (∆G ≈ 0 kJ/mol); **d** compound (IV) bearing four substituents (both nitro and thiocyanate groups) was capable of entering the catalytic cleft of the enzyme, however, none functional group was shown to interact with the catalytic amino acids. The compound demonstrated the higher positive score of the predicted Gibbs free energy (∆G ≈ + 2 kJ/mol). The hydrophobic surrounding of the catalytic cleft is mostly made up by following amino acids (shown as dots): Trp285, Ile288, Trp297, Val309, Gln310; Phe311; Thr315; Trp316; Trp349; Ala351; Trp352; Val354

With the object of discerning of the molecular basis to the observed distinctions in effectiveness of the compounds, the Flexx docking procedure [29], revealing the interaction of the BPT compounds with the active center of the RpfB molecule (*M. tuberculosis*, PDB 3EO5) was carried out (Fig. 3).

All the docked molecules lacking the nitro-substituents were potentially shown to interact with the catalytic cleft of the protein, being involved in hydrophobic interactions of their phenyl rings with Ile288, Phe311, Trp352, Val354, Trp285, Ala351, Val309, Trp349, Thr315 similarly with NPTs [11].

The results showed the predicted scores of the Gibbs free energy of interaction with the catalytic domain of RpfB ranging from − 10 to − 7 kJ/mol for (III), (II), (I); and about − 2.0 to 0.0 kJ/mol for the compounds with a maximal number of the substituents (VI), (V), evidencing conformity with the experimental data on supression of the enzymatic activity (Table 1).

Benzoylphenyl thiocyanate compounds were shown to interact with the catalytic cleft mainly through hydrophobic interactions, whereby the thiocyanate group was potentially able to interact with Asp312 (Fig. 3) as it was demonstrated in the previously published paper for (I) [11].

Obviously, the molecules with the extended number of the functional groups such as (IV) are unable to interact with the catalytic cleft by their functional groups (Fig. 3d), and all the interactions could be attributed to the interactions of the phenyl groups with the hydrophobic surroundings. The latter is confirmed by quenching of Rpf’s autofluorescence by the compound (IV) (Additional file 1: Figure S1), similar to the previous findings typical for NPTs, revealing the ability to interact with the active center through the phenyl rings only [10, 11].

## Conclusions

The present study demonstrates dispensability of the nitro group in the NPT’s core for inhibition of both the enzymatic and biological activities of the Rpf molecules. These findings should be taken into account in the development of new potential scaffolds capable of suppressing reactivation of the dormant mycobacterial cells.

## Additional file

**Additional file 1.** Results of fluorescence quenching of RpfB by bis(4-thiocyano-3-nitrophenyl)metanone (IV). Results are shown in the Stern-Volmer plot and in the modified Stern-Volmer plot (inset). The steady-state fluorescence excitation and emission spectra of RpfB protein were recorded at room temperature on the RF-5301PC fluorimeter (“SHIMADZU”, Japan) in 3×3 mm path length quartz cuvette; slit widths 1.5 mm (excitation) and 3 mm (emission). RpfB protein (25.6 μg/ml) was dissolved in 50 mM phosphate buffer, pH 6.0. The fluorescence emission spectra were recorded under excitation 280 nm [10]. The calculated accessibility of tryptophan residues for bis(4-thiocyano-3-nitrophenyl)metanone (IV) was close to 71 %, corresponding to interaction with three tryptophan residues out of five.

## References

[1] Parrish NM, Dick JD, Bishai WR (1998). Mechanisms of latency in *Mycobacterium tuberculosis*. Trends Microbiol.

[2] Salina E, Ryabova O, Kaprelyants A, Makarov V (2014). New 2-thiopyridines as potential candidates for killing both actively growing and dormant *Mycobacterium tuberculosis* cells. Antimicrob Agents Chemother.

[3] Mukamolova GV, Kaprelyants AS, Young DI, Young M, Kell DB (1998). A bacterial cytokine. Proc Natl Acad Sci USA.

[4] Cohen-Gonsaud M, Keep NH, Davies AP, Ward J, Henderson B, Labesse G (2004). Resuscitation-promoting factors possess a lysozyme-like domain. Trends Biochem Sci.

[5] Cohen-Gonsaud M, Barthe P, Bagnéris C, Henderson B, Ward J, Roumestand C, Keep NH (2005). The structure of a resuscitation-promoting factor domain from *Mycobacterium tuberculosis* shows homology to lysozymes. Nat Struct Mol Biol.

[6] Ruggiero A, Tizzano B, Geerlof A, Pedone E, Pedone C, Wilmanns M, Berisio R (2007). Expression, purification, crystallization and preliminary X-ray crystallographic analysis of a resuscitation-promoting factor from Mycobacterium tuberculosis. Acta Crystallogr Sect F Struct Biol Cryst Commun.

[7] Ruggiero A, Tizzano B, Pedone E, Pedone C, Wilmanns M, Berisio R (2009). Crystal structure of the resuscitation-promoting factor (DeltaDUF)RpfB from *M. tuberculosis*. J Mol Biol.

[8] Mukamolova GV, Murzin AG, Salina EG, Demina GR, Kell DB, Kaprelyants AS, Young M (2006). Muralitic activity of *Micrococcus luteus* Rpf and its relationship to physiological activity in promoting bacterial growth and resuscitation. Mol Microbiol.

[9] Telkov MV, Demina GR, Voloshin SA, Salina EG, Dudik TV, Stekhanova TN, Mukamolova GV, Kazaryan KA, Goncharenko AV, Young M, Kaprelyants AS (2006). Proteins of the Rpf (resuscitation promoting factor) family are peptidoglycan hydrolases. Biochemistry (Mosc).

[10] Demina GR, Makarov VA, Nikitushkin VD, Ryabova OB, Vostroknutova GN, Salina EG, Shleeva MO, Goncharenko AV, Kaprelyants AS (2009). Finding of the low molecular weight inhibitors of resuscitation promoting factor enzymatic and resuscitation activity. PLoS ONE.

[11] Ruggiero A, Marchant J, Squeglia F, Makarov V, De Simone A, Berisio R (2013). Molecular determinants of inactivation of the resuscitation promoting factor B from *Mycobacterium tuberculosis*. J Biomol Struct Dyn.

[12] Connell ND (1994). Mycobacterium: isolation, maintenance, transformation, and mutant selection. Methods Cell Biol.

[13] Clinical and Laboratory Standards Institute. Methods for dilution antimicrobial susceptibility tests for bacteria that grow aerobically; approved standard—Ninth edition. CLSI document M07-A9.

[14] Shleeva MO, Kudykina YK, Vostroknutova GN, Suzina NE, Mulyukin AL, Kaprelyants AS (2011). Dormant ovoid cells of *Mycobacterium tuberculosis* are formed in response to gradual external acidification. Tuberculosis (Edinb.).

[15] de Man JC (1975). The probability of most probable numbers. Eur J Appl Microbiol.

[16] Nikitushkin VD, Demina GR, Shleeva MO, Guryanova SV, Ruggiero A, Berisio R, Kaprelyants AS (2015). A product of RpfB and RipA joint enzymatic action promotes the resuscitation of dormant mycobacteria. FEBS J.

[17] Mahapatra S, Crick DC, McNeil MR, Brennan PJ (2008). Unique structural features of the peptidoglycan of *Mycobacterium leprae*. J Bacteriol.

[18] Hett EC, Chao MC, Rubin EJ (2010). Interaction and modulation of two antagonistic cell wall enzymes of mycobacteria. PLoS Pathog.

[19] Ruggiero A, Squeglia F, Pirone L, Correale S, Berisio R (2011). Expression, purification, crystallization and preliminary X-ray crystallographic analysis of a major fragment of the resuscitation-promoting factor RpfB from *Mycobacterium tuberculosis*. Acta Crystallogr Sect F Struct Biol Cryst Commun.

[20] Nikitushkin VD, Demina GR, Shleeva MO, Kaprelyants AS (2013). Peptidoglycan fragments stimulate resuscitation of “non-culturable” mycobacteria, Antonie van Leeuwenhoek. Int J Gen Mol Microbiol.

[21] Mukamolova GV, Turapov OA, Kazarian K, Telkov M, Kaprelyants AS, Kell DB, Young M (2002). The rpf gene of *Micrococcus luteus* encodes an essential secreted growth factor. Mol Microbiol.

[22] Tufariello JM, Jacobs WR, Chan J (2004). Individual *Mycobacterium tuberculosis* resuscitation-promoting factor homologues are dispensable for growth in vitro and in vivo. Infect Immun.

[23] Downing KJ, Betts JC, Young DI, McAdam RA, Kelly F, Young M, Mizrahi V (2004). Global expression profiling of strains harbouring null mutations reveals that the five rpf-like genes of *Mycobacterium tuberculosis* show functional redundancy. Tuberculosis (Edinb).

[24] Downing KJ, Mischenko VV, Shleeva MO, Young DI, Young M, Kaprelyants AS, Apt AS, Mizrahi V (2005). Mutants of *Mycobacterium tuberculosis* lacking three of the five rpf-like genes are defective for growth in vivo and for resuscitation in vitro. Infect Immun.

[25] Kana BD, Gordhan BG, Downing KJ, Sung N, Vostroktunova G, Machowski EE, Tsenova L, Young M, Kaprelyants A, Kaplan G, Mizrahi V (2008). The resuscitation-promoting factors of *Mycobacterium tuberculosis* are required for virulence and resuscitation from dormancy but are collectively dispensable for growth in vitro. Mol Microbiol.

[26] Hett EC, Rubin EJ (2008). Bacterial growth and cell division: a mycobacterial perspective. Microbiol Mol Biol Rev.

[27] Machowski EE, Senzani S, Ealand C, Kana BD (2014). Comparative genomics for mycobacterial peptidoglycan remodelling enzymes reveals extensive genetic multiplicity. BMC Microbiol.

[28] Filippova EV, Kieser KJ, Luan CH, Wawrzak Z, Kiryukhina O, Rubin EJ, Anderson WF (2016). Crystal structures of the transpeptidase domain of the *Mycobacterium tuberculosis* penicillin-binding protein PonA1 reveal potential mechanisms of antibiotic resistance. FEBS J.

[29] Rarey M, Kramer B, Lengauer T, Klebe G (1996). A fast flexible docking method using an incremental construction algorithm. J Mol Biol.

