# Supplementary material for: Benzoylphenyl thiocyanates are new, effective inhibitors of the mycobacterial resuscitation promoting factor B protein
Source: Ann Clin Microbiol Antimicrob. 2017 Nov 2;16:69. doi: 10.1186/s12941-017-0244-7 (PMC5667462; doi:10.1186/s12941-017-0244-7)
Supplement: Supplementary file 1 — Additional file 1. Results of fluorescence quenching of RpfB by bis(4-thiocyano-3-nitrophenyl)metanone (IV). Results are shown in the Stern-Volmer plot and in the modified Stern-Volmer plot (inset). The steady-state fluorescence excitation and emission spectra of RpfB protein were recorded at room temperature on the RF-5301PC fluorimeter (“SHIMADZU”, Japan) in 3×3 mm path length quartz cuvette; slit widths 1.5 mm (excitation) and 3 mm (emission). RpfB protein (25.6 μg/ml) was dissolved in 50 mM phosphate buffer, pH 6.0. The fluorescence emission spectra were recorded under excitation 280 nm [10]. The calculated accessibility of tryptophan residues for bis(4-thiocyano-3-nitrophenyl)metanone (IV) was close to 71 %, corresponding to interaction with three tryptophan residues out of five. [file 12941_2017_244_MOESM1_ESM.pdf]

Additional file 1

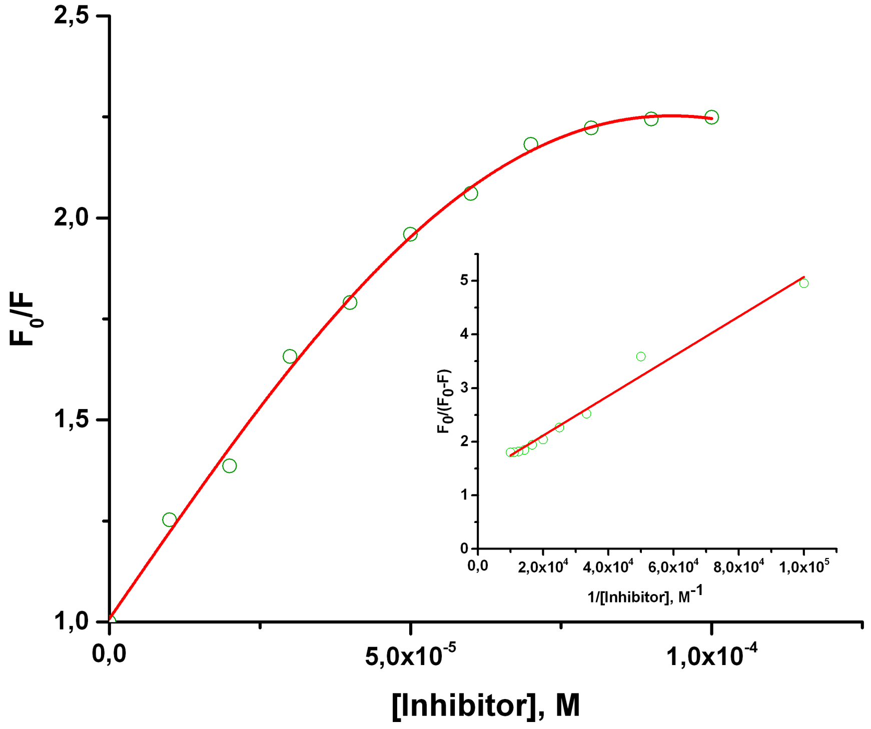

Figure S1. Results of fluorescence quenching of RpfB by bis(4-thiocyano-3-nitrophenyl)metanone (IV). Results are shown in the Stern-Volmer plot and in the modified Stern-Volmer plot (inset). The steady-state fluorescence excitation and emission spectra of RpfB protein were recorded at room temperature on the RF-5301PC fluorimeter ("SHIMADZU", Japan) in 3×3 mm path length quartz cuvette; slit widths 1.5 mm (excitation) and 3 mm (emission). RpfB protein (25.6 µg/ml) was dissolved in 50 mM phosphate buffer, pH 6.0. The fluorescence emission spectra were recorded under excitation 280 nm [10]. The calculated accessibility of tryptophan residues for bis(4-thiocyano-3-nitrophenyl)metanone (IV) was close to 71 %, corresponding to interaction with three tryptophan residues out of five.
